# Precursor Engineering of the Electron Transport Layer for Application in High‐Performance Perovskite Solar Cells

**DOI:** 10.1002/advs.202102845

**Published:** 2021-10-11

**Authors:** Zhichao Lin, Wenqi Zhang, Qingbin Cai, Xiangning Xu, Hongye Dong, Cheng Mu, Jian‐Ping Zhang

**Affiliations:** ^1^ Department of Chemistry Renmin University of China Beijing 100872 P. R. China

**Keywords:** charge transfer, doping, electron transport layers, perovskite solar cells, precursors

## Abstract

The electron transport layer (ETL) is a key component of regular perovskite solar cells to promote the overall charge extraction efficiency and tune the crystallinity of the perovskite layer for better device performance. The authors present a novel protocol of ETL engineering by incorporating a composition of the perovskite precursor, methylammonium chloride (MACl), or formamidine chloride (FACl), into SnO_2_ layers, which are then converted into the crystal nuclei of perovskites by reaction with PbI_2_. The SnO_2_‐embedded nuclei remarkably improve the morphology and crystallinity of the optically active perovskite layers. The improved ETL‐to‐perovskite electrical contact and dense packing of large‐grained perovskites enhance the carrier mobility and suppress charge recombination. The power conversion efficiency increases from 20.12% (blank device) to 21.87% (21.72%) for devices with MACl (FACl) as an ETL dopant. Moreover, all the precursor‐engineered cells exhibit a record‐high fill factor (82%).

## Graphical abstract

Methylammonium chloride (MACl) or formamidine chloride (FACl) of perovskite precursor is added into SnO_2_ electron transport layer (ETL) and reacts with PbI_2_ to form perovskite crystal nucleus, which improves the contact between SnO_2_ ETL and perovskite, as well as the morphology and crystallinity of perovskite layer, and greatly improves the fill factor (FF) of perovskite solar cells (PSCs).

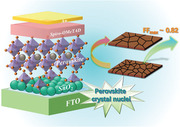

## 1 Introduction

Perovskite solar cells (PSCs) have been developed as a novel type of solar cell with significant potential in the field of advanced photovoltaic technology.^[^ ^1^, ^2^, ^3^ ^]^ Their power conversion efficiency (PCE) has rapidly improved over the past decade, increasing from 3.8% at their inception to over 25% at present.^[^ ^4^, ^5^, ^6^, ^7^ ^]^ The electron transport layer (ETL) in PSCs plays an indispensable role in transporting electrons, while simultaneously blocking holes. In a regular n‐i‐p type PSC of planar configuration, the structure of the ETL has a significant effect on the morphology and crystallinity of its neighboring perovskite layers.^[^ ^8^, ^9^, ^10^, ^11^, ^12^, ^13^, ^14^, ^15^, ^16^, ^17^, ^18^, ^19^ ^]^ Therefore, considerable research has been devoted to improving the PSC performance via ETL modification.^[^ ^20^, ^21^, ^22^, ^23^, ^24^, ^25^, ^26^, ^27^, ^28^, ^29^, ^30^, ^31^ ^]^ SnO_2_ nanocrystals are a widely used ETL material that can be oriented so as to facilitate the vertical growth of the perovskite crystals, a morphology essential to charge carrier transport. In addition, a suitable structural modification of the SnO_2_ ETL can result in an enhanced ETL‐perovskite interfacial contact as well as increased pack densification and grain size of the perovskite crystals, which, in turn, increase the fill factor (FF) and stability of the PSC devices.^[^ ^32^, ^33^ ^]^

The additive engineering of perovskite precursors has been performed extensively to increase the crystallinity of optically active perovskite layers and hence, improve the device performance.^[^ ^34^, ^35^, ^36^, ^37^, ^38^, ^39^, ^40^ ^]^ In this regard, Kim et al. investigated the addition of a methylammonium chloride (MACl) precursor to a formamidinium lead iodide (FAPbI_3_) perovskite, and reported a significant improvement in the quality of the resultant perovskite films in terms of an increased grain size, crystallinity, and photoluminescence lifetime.^[^ ^34^ ^]^ Zhou et al. proposed a perovskite formation mechanism using a formamidine chloride (FACl) dopant, which effectively increased the size and crystallinity of the perovskite crystallites.^[^ ^35^ ^]^ Although perovskite precursors have proven effective in optimizing the performance of optically active layers, their application in SnO_2_ ETLs has been rarely reported in the literature.^[^ ^41^ ^]^

Herein, we report a facile and effective process for ETL modification through the addition of a perovskite precursor. Initially, two predetermined precursor compositions, specifically MACl and FACl, were introduced into the SnO_2_ layers. These dopants facilitated the nucleation of perovskite nuclei embedded within the ETLs, leading to an improvement in the quality of the perovskite layers while also increasing the SnO_2_‐perovskite contact. In comparison to the PCE exhibited by the undoped device (20.12%), the MACl‐ and FACl‐doped devices exhibited improved PCEs of 21.87% and 21.72%, respectively. The improvement in the photovoltaic performance of these PSCs was attributed to the perovskite precursor engineering of the ETLs, which imparted enhanced perovskite film morphology, reduced free charge carrier trap density, and increased FF. This study practically demonstrates the effectiveness of the perovskite precursor engineering of the ETL to significantly improve the performance of PSCs.

## 2 Results and Discussion

**Figure 1:**
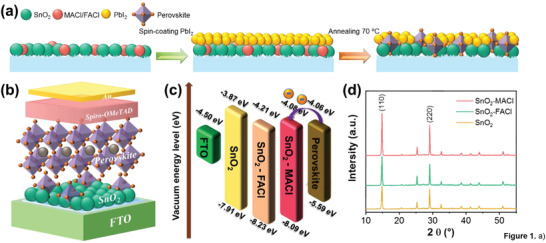
a) Schematic showing the preparation of the SnO_2_‐MACl/FACl thin films. b) Schematic of the fabricated SnO_2_‐MACl/FACl device. c) Energy‐level diagram of a typical PSC. d) XRD spectra of the perovskite films deposited on the SnO_2_‐MACl, SnO_2_‐FACl, and pure SnO_2_ ETLs.

**Figure** **1**a shows a schematic detailing the process of embedding perovskite crystal nuclei into the ETL. First, the mixed SnO_2_‐MACl/SnO_2_‐FACl solution was spin‐coated onto the fluorine‐doped tin oxide (FTO) substrate surface and annealed. Scanning electron microscopy (SEM) images obtained from the SnO_2_‐MACl and SnO_2_‐FACl films are shown in Figure S1, Supporting Information. The ETLs doped with either MACl or FACl exhibited increased densification surface morphology, which promoted the development of perovskite grains.^[^ ^42^ ^]^ Subsequently, the sample surfaces were coated with PbI_2_ and annealed at 70 °C. During annealing, PbI_2_ reacts with the MACl or FACl present in the ETL to form perovskite crystal nuclei. As shown in Figure S2, Supporting Information, the samples doped with MACl or FACl (Figure S2a,b, Supporting Information) exhibited a significantly larger grain size than the undoped samples (Figure S2c, Supporting Information), suggesting the formation of perovskite grains in the doped samples. The X‐ray diffraction (XRD) spectrum (Figure S3, Supporting Information) exhibits a peak at 31.89°, which corresponds to that of a perovskite crystal, confirming its formation.^[^ ^43^, ^44^, ^45^ ^]^ A schematic of the SnO_2_‐MACl/FACl ETL device is shown in Figure 1b. The perovskite crystal nucleus was embedded in the SnO_2_ ETL, which promoted the formation and development of perovskite particles.

The effect of MACl/FACl on the surface energy of the SnO_2_ ETLs was evaluated using ultraviolet photoelectron spectroscopy (UPS) and ultraviolet–visible spectroscopy (UV–Vis). The Fermi level (*E*_F_), valence band (*E*_VB_), and conduction band (*E*_CB_) were obtained directly from the recorded UPS and UV–Vis spectra (Figure S4, Supporting Information). In accordance with the energies shown in Table S1, Supporting Information, the energy‐level diagram of each device is plotted in Figure 1c. The spectra generated by each of the three ETLs exhibited significant variations. In comparison with the pure SnO_2_ ETL, the *E*_CB_ values exhibited by the SnO_2_‐MACl/FACl ETLs were close to that of a perovskite film, a property which is beneficial during the extraction and transfer of photogenerated electrons from the perovskite film to the SnO_2_‐MACl/FACl ETLs.

**Figure 2:**
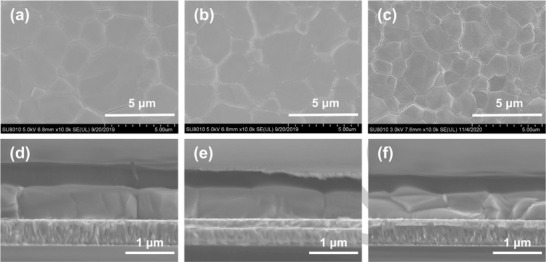
SEM images obtained from perovskite films deposited on a) SnO_2_‐MACl, b) SnO_2_‐FACl, and c) pure SnO_2_ films. Cross‐sectional SEM images of typical PSCs containing d) SnO_2_‐MACl, e) SnO_2_‐FACl, and f) pure SnO_2_ ETLs.

To study the effect of a MACl/FACl‐doped ETL on the crystallinity of perovskite films, the perovskite films derived from the SnO_2_‐MACl, SnO_2_‐FACl, and pure SnO_2_ ETL substrates were analyzed using XRD. As shown in Figure 1d and Figure S5, Supporting Information, the diffraction peaks corresponding to the (110) and (220) planes of the perovskite films are significantly stronger and sharper in the spectra of the SnO_2_‐MACl/FACl‐containing ETL samples in comparison to those of the pure SnO_2_ ETL sample, and the full width at half maximum values of the (110) peak are reduced (Table S2, Supporting Information). This result suggests that the crystallinity of the perovskite films was significantly enhanced upon doping with MACl/FACl.^[^ ^46^ ^]^ The above results were consistent with the SEM characterization results shown in **Figure** **2**.

The morphologies of the perovskite films obtained using the SnO_2_‐MACl, SnO_2_‐FACl, and pure SnO_2_ ETL substrates were evaluated using SEM. In comparison with the perovskite films containing a pure SnO_2_ ETL (Figure 2c), the perovskite films containing SnO_2_‐MACl/FACl ETLs (Figure 2a,b) exhibited a significantly larger grain size, with a maximum value of 5 µm. An increase in the grain size will lead to a decrease in the grain boundary concentration, thereby reducing the capacity for carrier recombination at grain boundaries and nonradiative recombination loss.^[^ ^47^ ^]^ According to the nucleation theory, the growth of the perovskite film consists of four processes: the first step is to generate the crystal nuclei; the second step is to grow into an island structure; the third step further evolves into the network structure; and the last step is to form a continuous layer.^[^ ^13^ ^]^ In the samples modified by MACl and FACl, perovskite nuclei have been formed in the process of spin coating PbI_2_ and annealing. Therefore, it can directly evolve into island structure on the basis of existing nuclei, form a networked microstructure, and grow into continuous films. Figure 2d–f shows cross‐sectional SEM images obtained from the three PSCs containing pure SnO_2_, SnO_2_‐MACl, and SnO_2_‐FACl ETLs. The perovskite particles that developed on the pure SnO_2_ ETL exhibited a random distribution with respect to their orientation (Figure 2f). However, the single grains developed on the perovskite films obtained using SnO_2_‐MACl/FACl ETLs were oriented vertically with respect to both, the ETL and hole transport layer (HTL) (Figure 2d,e), a configuration which is conducive to charge transmission in PSCs.^[^ ^32^, ^33^ ^]^

**Figure 3:**
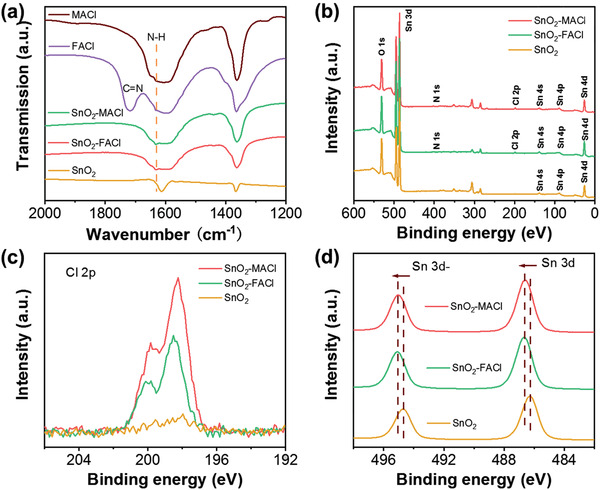
a) IR spectra of the MACl, FACl, SnO_2_‐MACl, and SnO_2_‐FACl samples, b) XPS profiles of the SnO_2_‐MACl, SnO_2_‐FACl, and pure SnO_2_ films, c) high‐resolution Cl 2p XPS profiles of the SnO_2_‐MACl, SnO_2_‐FACl, and pure SnO_2_ films, and d) high‐resolution Sn 3d XPS profiles of the SnO_2_‐MACl, SnO_2_‐FACl, and pure SnO_2_ films.

The extent of the interaction between SnO_2_ and the MACl/FACl dopants was evaluated using infrared (IR) spectroscopy and X‐ray photoelectron spectroscopy (XPS). In the IR spectra shown in **Figure** **3**a, a peak occurs at 1640 cm^−1^ in both, the SnO_2_‐MACl and SnO_2_‐FACl samples. This peak corresponds to the vibration of the N—H bond, which is related to the presence of MA^+^/FA^+^ ions.^[^ ^48^ ^]^ However, comparing the IR spectra of FACl and SnO_2_‐FACl, it was found that the characteristic transmission band of C═N originally appeared at 1720 cm^−1^ in FACl samples disappeared in SnO_2_‐FACl samples, indicating that FA^+^ ions bind to hydroxyl groups on SnO_2_ surface through C═N bonds.^[^ ^41^, ^48^ ^]^ XPS curve of O1s level in Figure S6, Supporting Information, also proves the above phenomenon. The XPS spectrum of O1s of all samples can be deconvoluted into two different peaks, corresponding to the hydroxyl group (OH^−^) on the surface of SnO_2_ (532.2 eV) and the saturated oxygen (O^2−^) in SnO_2_ (530.3 eV).^[^ ^12^ ^]^ It can be seen that the ratio of OH^−^ peak to O^2−^ peak of SnO_2_‐MACl sample is only slightly lower than that of pure SnO_2_ sample, while the ratio of SnO_2_‐FACl samples decreased significantly,^[^ ^12^ ^]^ which are consistent with the results of SEM image of Figure S1, Supporting Information. The survey XPS profiles in Figure 3b confirm the presence of N, Cl, Sn, and O in the SnO_2_‐MACl/FACl samples.^[^ ^49^, ^50^ ^]^ Figure 3c compares the Cl 2p XPS profiles of the SnO_2_‐MACl, SnO_2_‐FACl, and pure SnO_2_ samples. The results suggest that SnO_2_ was successfully modified through the addition of MACl/FACl. The Sn 3d XPS profiles in Figure 3d show that the Sn 3d double peaks arising from the SnO_2_‐MACl and SnO_2_‐FACl samples undergo a shift to a higher photoelectron binding energy in comparison to the pure SnO_2_ sample, suggesting that the introduction of Cl in the sample induced a negative charge in the vicinity of the Sn atom.^[^ ^42^, ^50^ ^]^ Figure S7, Supporting Information, shows the UV–Vis absorption spectra of perovskite films deposited on SnO_2_‐MACl, SnO_2_‐FACl, and pure SnO_2_ ETLs. It can be seen that the addition of MACl and FACl will not affect the absorption edge and absorption intensity of perovskite films.

**Figure 4:**
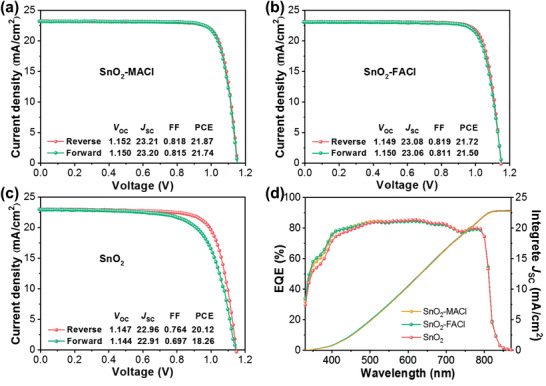
Optimal *J*–*V* curves of the typical PSCs obtained using the a) SnO_2_‐MACl, b) SnO_2_‐FACl, and c) pure SnO_2_ ETLs. d) EQE spectra of the typical PSCs obtained using the SnO_2_‐MACl, SnO_2_‐FACl, and pure SnO_2_ ETLs.

To study the effect of the MACl and FACl dopants on electron transfer in the PSCs, the current density–voltage (*J*–*V*) trend exhibited by each sample was evaluated. **Figure** **4**a–c shows the optimal *J*–*V* performance exhibited by each of the three devices. The SnO_2_‐MACl‐ and SnO_2_‐FACl‐based PSCs exhibited reverse‐scan PCEs of up to 21.87% and 21.72%, respectively, significantly higher than the PCE of the pure SnO_2_ PSC (20.12%). This was primarily attributed to a significant increase in both, the FF from 0.764 to 0.818 and 0.819, and the open‐circuit voltage (*V*_OC_) from 1.147 to 1.152 and 1.149 V in the SnO_2_‐MACl‐ and SnO_2_‐FACl‐based PSCs, respectively. In addition, the short‐circuit current density (*J*_SC_) increased from 22.96 to 23.21 and 23.08 mA cm^−2^ in the SnO_2_‐MACl‐ and SnO_2_‐FACl‐based PSCs, respectively. Further, the SnO_2_‐MACl‐ and SnO_2_‐FACl‐based ETLs essentially eliminate any hysteresis, suggesting that both, the SnO_2_‐MACl and SnO_2_‐FACl ETLs, exhibit stronger charge transfer capabilities than the pure SnO_2_ ETL. Figure 4d shows the external quantum efficiency (EQE) spectra of the SnO_2_‐MACl, SnO_2_‐FACl, and pure SnO_2_ ETL PSCs. Analysis of these spectra revealed that the integrated current densities of the three PSCs were 22.87, 22.79, and 22.74 mA cm^−2^, respectively, values which were consistent with the *J*–*V* measurement results. Concurrently, the stabilized photocurrent output of PSCs with the SnO_2_‐MACl, SnO_2_‐FACl, and single SnO_2_ ETLs were recorded at the maximum power point voltage (Figure S8, Supporting Information). Steady‐state PCEs of 20.8%, 20.3%, and 18.9% were respectively obtained.

**Figure 5:**
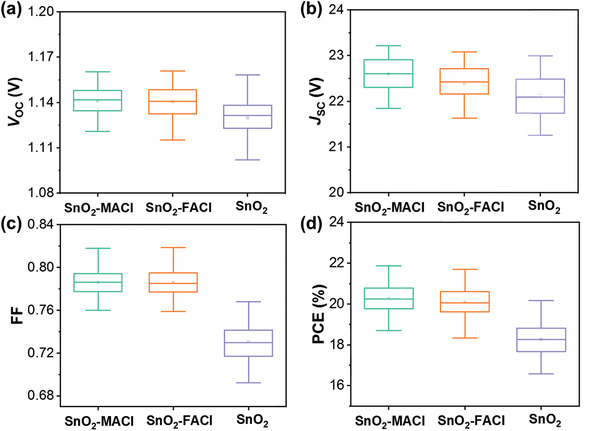
Statistical distributions of the a) *V*_OC_, b) *J*_SC_, c) FF, and d) PCE values of typical PSCs based on the SnO_2_‐MACl, SnO_2_‐FACl, and pure SnO_2_ ETLs (data collected from 30 cells).

**Figure** **5**a–d shows the statistical distribution of the *V*_OC_, *J*_SC_, FF, and PCE values of each of the PSC samples. The average *V*_OC_ values exhibited by the PSCs based on SnO_2_‐MACl and SnO_2_‐FACl ETLs were 1.141 and 1.140 V, respectively, which were higher than those exhibited by PSCs based on a pure SnO_2_ ETL (1.130 V). In addition, the average *J*_SC_ values exhibited by the PSCs based on SnO_2_‐MACl and SnO_2_‐FACl ETLs were 22.59 and 22.39 mA cm^−2^, respectively, which were higher than those exhibited by PSCs based on a pure SnO_2_ ETL (22.13 mA cm^−2^). The average FFs exhibited by the PSCs based on SnO_2_‐MACl and SnO_2_‐FACl ETLs were 0.786 and 0.785, respectively, which were significantly higher than those exhibited by PSCs based on a single SnO_2_ ETL (0.730). Overall, the average PCEs of the PSCs based on SnO_2_‐MACl and SnO_2_‐FACl ETLs were 20.27% and 20.08%, respectively, which were significantly higher than those of the PSCs based on single SnO_2_ ETLs (18.37%). The improvement in the photovoltaic properties exhibited by the doped PSCs was attributed to the improvement in the surface morphology of the perovskite films. These improvements facilitated a reduction in the trap density, which imparted an increased FF in the corresponding PSCs.

**Figure 6:**
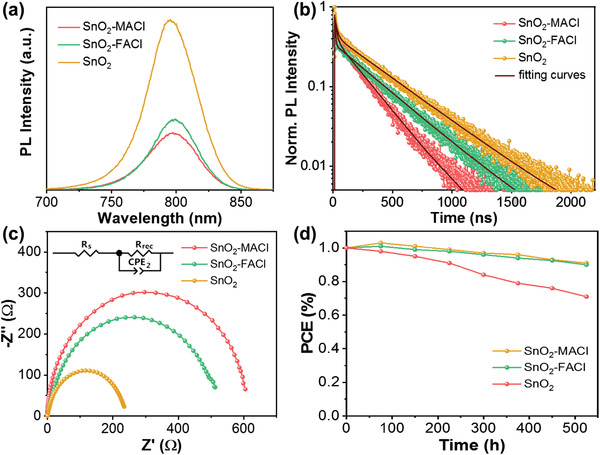
a) Steady‐state photoluminescence (PL) and b) time‐resolved photoluminescence (TRPL) spectra exhibited by perovskite films deposited on the SnO_2_‐MACl, SnO_2_‐FACl, and pure SnO_2_ ETLs. c) Nyquist plots obtained under dark conditions from the PSCs derived from the SnO_2_‐MACl, SnO_2_‐FACl, and pure SnO_2_ ETLs. d) Long‐term stability performance exhibited by the PSCs derived from the SnO_2_‐MACl, SnO_2_‐FACl, and pure SnO_2_ ETLs without encapsulation under ambient conditions.

Steady‐state photoluminescence (PL) and time‐resolved photoluminescence (TRPL) were used to evaluate the charge dynamics between the perovskite and ETL. In comparison to the pure SnO_2_ ETL, the PL intensities exhibited by the perovskite films deposited on the SnO_2_‐MACl/FACl‐based ETLs were significantly reduced (**Figure** **6**a).This result suggests that SnO_2_‐MACl/FACl‐based ETLs enhance electron extraction and transport at the interface. This result was further validated by the TRPL results shown in Figure 6b. The PL decay of the perovskite films deposited on SnO_2_‐MACl/FACl‐based ETLs occurred at an increased rate in comparison to the perovskite films deposited on pure SnO_2_ ETLs. The corresponding lifetimes of the perovskite films deposited on the SnO_2_‐MACl, SnO_2_‐FACl, and single SnO_2_ ETLs were 150, 184, and 273 ns, respectively.

The charge transfer mechanisms of the PSCs were investigated further via electrochemical impedance spectroscopy (EIS). The resultant Nyquist plots (Figure 6c) were obtained by placing the PSCs in dark conditions under a 5 V bias, while the contact resistance (*R*_co_) and recombination resistance (*R*_rec_) were obtained by fitting the recorded results (Table S3, Supporting Information). The reduced *R*_co_ values exhibited by PSCs utilizing SnO_2_‐MACl/FACl‐based ETLs suggest that SnO_2_‐MACl/FACl enhanced the charge transfer ability of the cells in comparison to the pure SnO_2_ samples.^[^ ^42^, ^50^ ^]^

To investigate the stability of the PSC devices, a sample of each composition was stored in a glove box to simulate the aging process. Figure 6d shows the resultant PCEs of all three PSCs. Although the PSCs stored in the glove box were not susceptible to corrosion by oxygen and water, they were still affected by interface diffusion, ion migration, and electrode corrosion. After an aging period of 500 h, the PSCs based on the SnO_2_‐MACl/FACl ETLs exhibited efficiencies of 90% with respect to their original values, while those based on a pure SnO_2_ ETL exhibited efficiencies of just 70% with respect to their original values. These results suggest that the stability of PSCs based on SnO_2_‐MACl/FACl ETLs is significantly higher than that of their pure SnO_2_ ETL counterparts.

## 3 Conclusion

In summary, a novel process for the modification of ETLs using perovskite precursors was proposed. MACl/FACl perovskite precursors were integrated into the ETL microstructures to form the perovskite nuclei. The perovskite crystal nuclei embedded within the ETL microstructures promoted the development of perovskite crystals, leading to the formation of perovskite films with larger grains and increased crystallinity. The thus‐formed dense and pore‐free films suppressed defect formation and improved the FF of the device, thereby improving the PCE of the corresponding PSC. The introduction of MACl or FACl into the ETLs significantly improved the FF of the PSCs, and from 0.764 to 0.818 and 0.819, respectively. The overall PCEs of the two PSCs doped with MACl and FACl were 21.87% and 21.72%, respectively. The PCEs recorded for both the doped PSCs were significantly higher than those recorded for the unmodified device (20.12%). The results of this study should provide a strong basis for the preparation of a PSC with enhanced performance and large perovskite grains, through the modification of the ETL.

## 4 Experimental Section

### Materials

FTO was purchased from OPVtech (China). Tin (IV) oxide (SnO_2_) nanoparticles (15% in H_2_O, colloidal dispersion) were purchased from Alfa Aesar. Lead (II) iodide (PbI_2_) (99.99%, trace metals basis), for use in the perovskite precursor, was obtained from Tokyo Chemical Industry Co., Ltd. Methylammonium chloride (CH_3_NH_3_Cl) (MACl) (99.5%, subjected to four rounds of purification), formamidinium chloride (HC(NH_2_)_2_Cl) (FACl) (99.5%, subjected to four rounds of purification), methylammonium bromide (CH_3_NH_3_Br) (MABr) (99.5%, subjected to four rounds of purification), and formamidinium iodide (HC(NH_2_)_2_I) (FAI) (99.5%, subjected to four rounds of purification) were purchased from Xi'an Polymer Light Technology Corp. 2,2′,7,7′‐tetrakis‐(*N,N*‐di‐p‐methoxyphenylamine)‐9,9′‐spirobifluorene (Spiro‐OMeTAD) (99.86%) was purchased from Advanced Election Technology. *N,N*‐dimethylformamide (DMF) (99.8%), dimethyl sulfoxide (DMSO) (99.9%), chlorobenzene (CB) (99.8%), 4‐tert‐butylpyridine (96%), and lithium bis (trifluoromethylsulfonyl) imide (99%) were purchased from J & K (China) Co., Ltd. Isopropyl alcohol (IPA) (99.9%) was obtained from Energy Chemical. Cobalt (III) FK209 TFSI salt (99.9%) was obtained from Greatcell Solar Materials Pty. Ltd. None of the reagents was purified further prior to their use.

### Device Fabrication

The FTO glass was sequentially cleaned using deionised water, acetone, and isopropanol in an ultrasonicator before being dried. Prior to use, the FTO glass was treated with oxygen plasma for 3 min. Subsequently, 500 mL of the SnO_2_ colloidal solution was mixed with 2500 mL of deionised water, and 0.2 mmol of both MACl or FACl were then added to the solution. Once a uniformly dispersed solution was obtained via stirring, the solution was spin‐coated onto the FTO surface at 4000 rpm for 30 s, and then the MACl modified substrate and FACl modified substrate were annealed for 30 min at 120 and 150 °C, respectively, to produce the ETL. Subsequently, 1.5 m PbI_2_ was dissolved in a DMF‐DMSO (9:1 volume ratio) mixed solvent and stirred overnight. The homogeneous solvent was then spin‐coated onto the surface of the ETL at 1500 rpm for 30 s, prior to annealing at 70 °C for 1 min. Meanwhile, an FAI:MABr:MACl (270:27:27 mg in 3 mL IPA) mixed solution was prepared and spin‐coated onto the PbI_2_ surface at 2000 rpm for 30 s, prior to annealing at 150 °C for 15 min in ambient air (≈40% humidity) to form the perovskite layer. Subsequently, 73 mg of spiro‐OMeTAD was dissolved in 1 mL of CB. Following this, 16.8 µL of Li‐TFSI (520 mg mL^−1^ in acetonitrile), 27.6 µL of cobalt (III) FK209 (300 mg mL^−1^ in acetonitrile), and 27.6 µL of 4‐tert‐butylpyridine were added to the solution to prepare the final HTL precursor solution. The HTL precursor solution was then spin‐coated onto the perovskite film at 3000 rpm for 30 s. Finally, an 80 nm Au film was deposited on the outer layer of the device through thermal evaporation.

### Characterization

Scanning electron microscopy (SEM) (Hitachi, SU8010) was used to evaluate the microstructure of the ETLs and perovskite films. The crystal structure and mass of the PbI_2_ and perovskite films were characterized by X‐ray diffraction (Shimadzu XRD‐7000). UPS of the SnO_2_‐MACl, SnO_2_‐FACl, and single SnO_2_ films was performed using a He I (21.22 eV) excitation line (Thermo Scientific ESCALab 250Xi). UV–Vis absorption spectra of the SnO_2_‐MACl, SnO_2_‐FACl, and pure SnO_2_ films were obtained (Agilent Cary60). The MACl, FACl, SnO_2_‐MACl, and SnO_2_‐FACl samples were subjected to Fourier transform infrared (FTIR) analysis (IRPrestige‐21). The photoluminescence (PL) of the perovskite films deposited on the SnO_2_‐MACl, SnO_2_‐FACl, and pure SnO_2_ films was measured and recorded (Hitachi F‐4600, 470 nm excitation). Electrochemical impedance spectroscopy (EIS) was performed on the PSCs based on the SnO_2_‐MACl, SnO_2_‐FACl, and pure SnO_2_ ETLs, using a scanning frequency between 1 and 10^6^ Hz, and an AC amplitude of 5 mV (ZAHENR PP211). The external quantum efficiencies (EQEs) of the PSCs based on the SnO_2_‐MACl, SnO_2_‐FACl, and single SnO_2_ ETLs were obtained in the wavelength range of 330–870 nm (EnliTech QE‐R). The current density–voltage (*J*–*V*) characteristics of the PSCs derived from the SnO_2_‐MACl, SnO_2_‐FACl, and pure SnO_2_ ETLs were measured in air without encapsulation under AM 1.5G illumination (Keithley 2400 Source Meter), for which each of the PSCs were covered with a metal aperture mask to define an active area of 0.1 cm^2^.

## Conflict of Interest

The authors declare no conflict of interest.

## Supporting information

Supporting Information

## Data Availability

Research Data are not shared.
