# Supplementary material for: Precursor Engineering of the Electron Transport Layer for Application in High‐Performance Perovskite Solar Cells
Source: Adv Sci (Weinh). 2021 Oct 11;8(22):2102845. doi: 10.1002/advs.202102845 (PMC8596138; doi:10.1002/advs.202102845)
Supplement: Supplementary file 1 — Supporting Information [file ADVS-8-2102845-s001.pdf]

© 2021 Wiley-VCH GmbH

ADVANCED SCIENCE
Open Access

Supporting Information

Precursor Engineering of the Electron Transport Layer for Application in High-Performance Perovskite Solar Cells

Zhichao Lin, Wenqi Zhang, Qingbin Cai, Xiangning Xu, Hongye Dong, Cheng Mu*, and Jian-Ping Zhang*

© 2021 Wiley-VCH GmbH

Precursor Engineering of the Electron Transport Layer for Application in High-Performance Perovskite Solar Cells

Zhichao Lin*, Wenqi Zhang, Qingbin Cai, Xiangning Xu, Hongye Dong, Cheng Mu*, and Jian-Ping Zhang

Department of Chemistry, Renmin University of China, Beijing, 100872, P. R. China.

*Corresponding Author. E-mail: cmu@ruc.edu.cn (Cheng Mu); linzc@stu.shzu.edu.cn (Zhichao Lin).

Table S1. Band gaps (E_g), secondary-electron cut-off ($E_{cut\text{-}off}$), Fermi level (E_F), valence band (E_{VB}) and conduction band (E_{CB}) of SnO_2-MACl, SnO_2-FACl, and pure SnO_2 films.

Samples	E_g (eV)	$E_{cut\text{-}off}$ (eV)	E_F (eV)	E_{VB} (eV)	E_{CB} (eV)
SnO_2- MACl	4.01	16.45	4.77	8.09	4.08
SnO_2- FACl	4.02	16.37	4.85	8.23	4.21
SnO_2	4.04	16.77	4.45	7.91	3.87

The formula $E_F = E_{cut\text{-}off} - 21.22$ eV was used to obtain the Fermi level (E_F), where $E_{cut\text{-}off}$ is the cut-off binding energy, and 21.22 eV is the emission energy of He I irradiation. The $E_{cut\text{-}off}$ values of the SnO_2-MACl, SnO_2-FACl, and pure SnO_2 films were 16.45, 16.37 and 16.77 eV, respectively. The E_F values of the SnO_2-MACl, SnO_2-FACl, and pure SnO_2 films were determined as −4.77, −4.85 and −4.45 eV, respectively. The E_{VB} values of the SnO_2-MACl, SnO_2-FACl, and pure SnO_2 films were −8.09, −8.23 and −7.91 eV, respectively, and were calculated using the relation $E_{VB} = E_F - E_{F,\ edge}$ (Fermi edge). Analysis of the absorption spectra and corresponding Tauc plots allowed the determination of the band gaps (E_g) of the SnO_2-MACl, SnO_2-FACl, and pure SnO_2 films to be obtained (4.01, 4.02 and 4.04 eV, respectively). The E_{CB} values obtained using E_g and E_{VB} were −4.08, −4.21 and −3.87 eV, respectively.

Table S2. Full width at half maximum (FWHM) of the (110) peak in XRD of perovskite films deposited on the SnO_2-MACl, SnO_2-FACl, and pure SnO_2 films.

Samples	FWHM
SnO_2- MACl	0.235
SnO_2- FACl	0.258
SnO_2	0.276

© 2021 Wiley-VCH GmbH

Table S3. Various resistivity values for each sample obtained under dark conditions during EIS.

Samples	R_s (Ω)	R_{rec} (Ω)
SnO_2- MACl	0.08	631.70
SnO_2- FACl	0.10	539.40
SnO_2	0.10	248.30

© 2021 Wiley-VCH GmbH

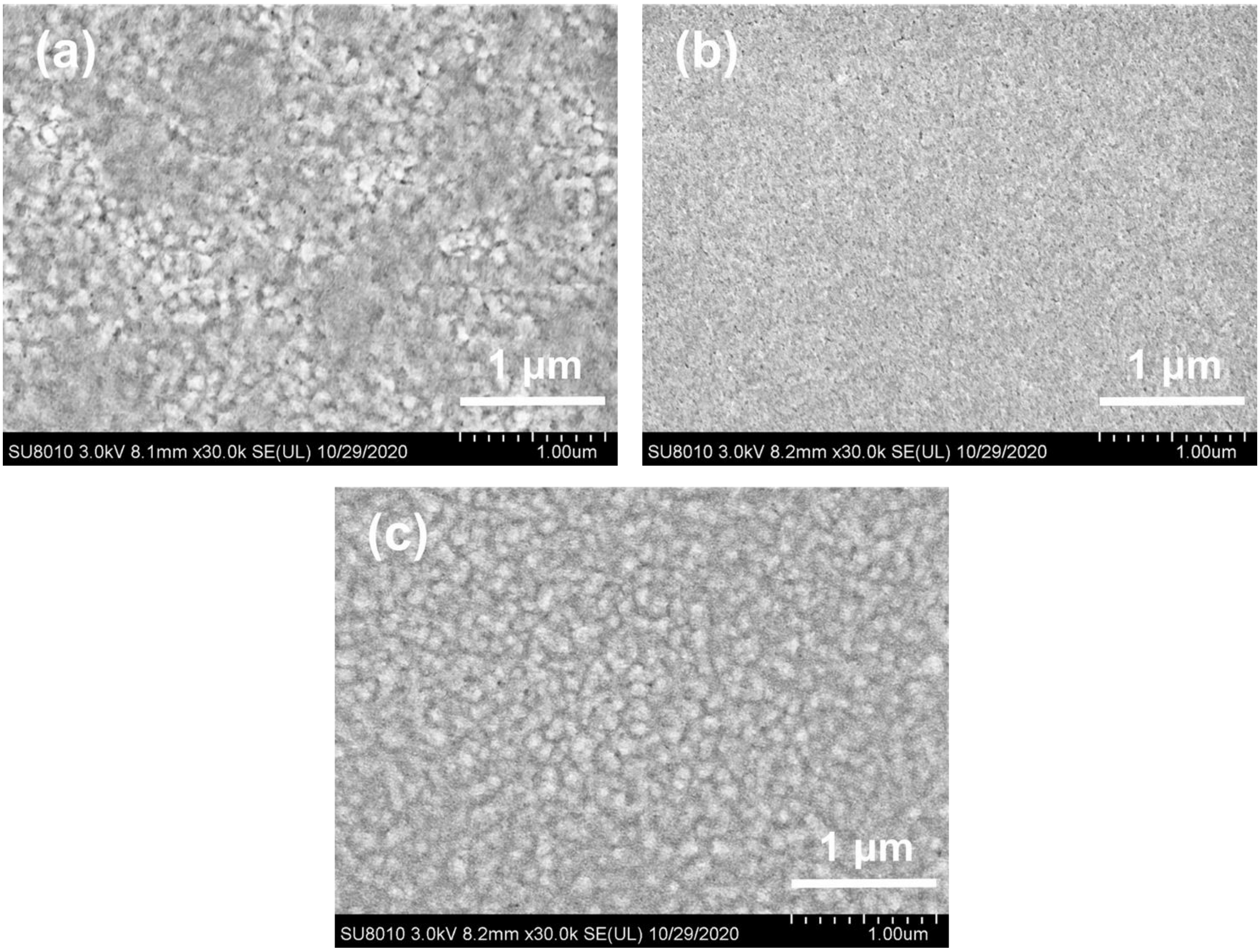

Figure S1. SEM images of a) SnO_2-MACl, b) SnO_2-FACl, and c) pure SnO_2 films deposited on the FTO/MgO substrate.

© 2021 Wiley-VCH GmbH

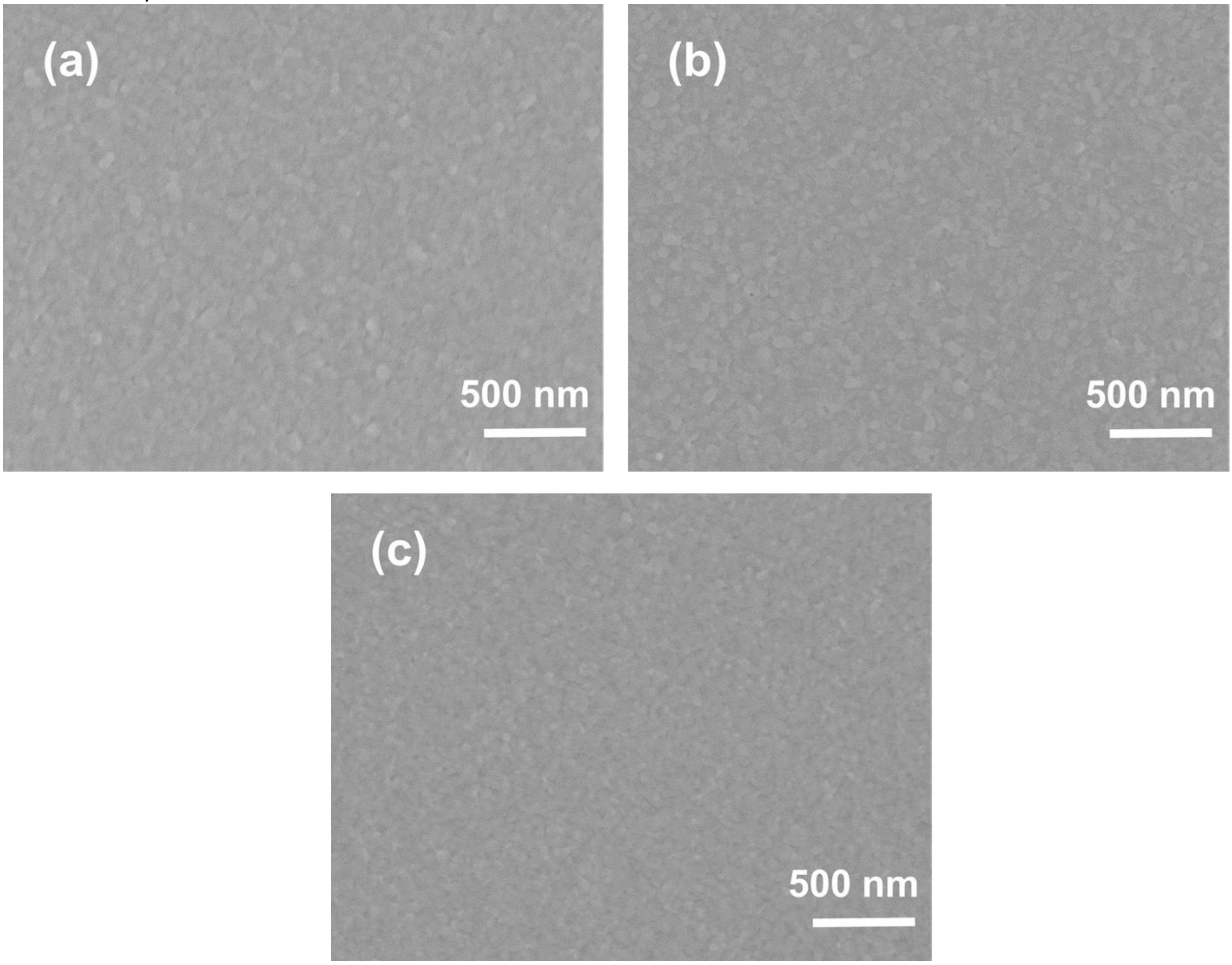

Figure S2. SEM images of the PbI_2 layer deposited on a) SnO_2-MACl, b) SnO_2-FACl, and c) pure SnO_2 films. Each sample was annealed at 70 °C.

© 2021 Wiley-VCH GmbH

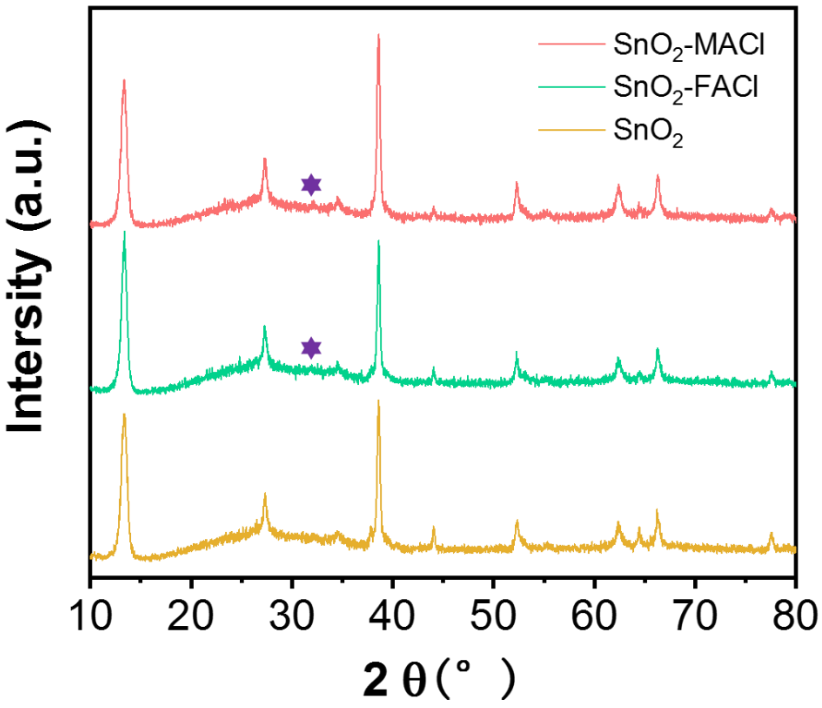

Figure S3. XRD patterns of the PbI_2 layer deposited on the SnO_2-MACl, SnO_2-FACl, and pure SnO_2 films. Each sample was annealed at 70 °C.

© 2021 Wiley-VCH GmbH

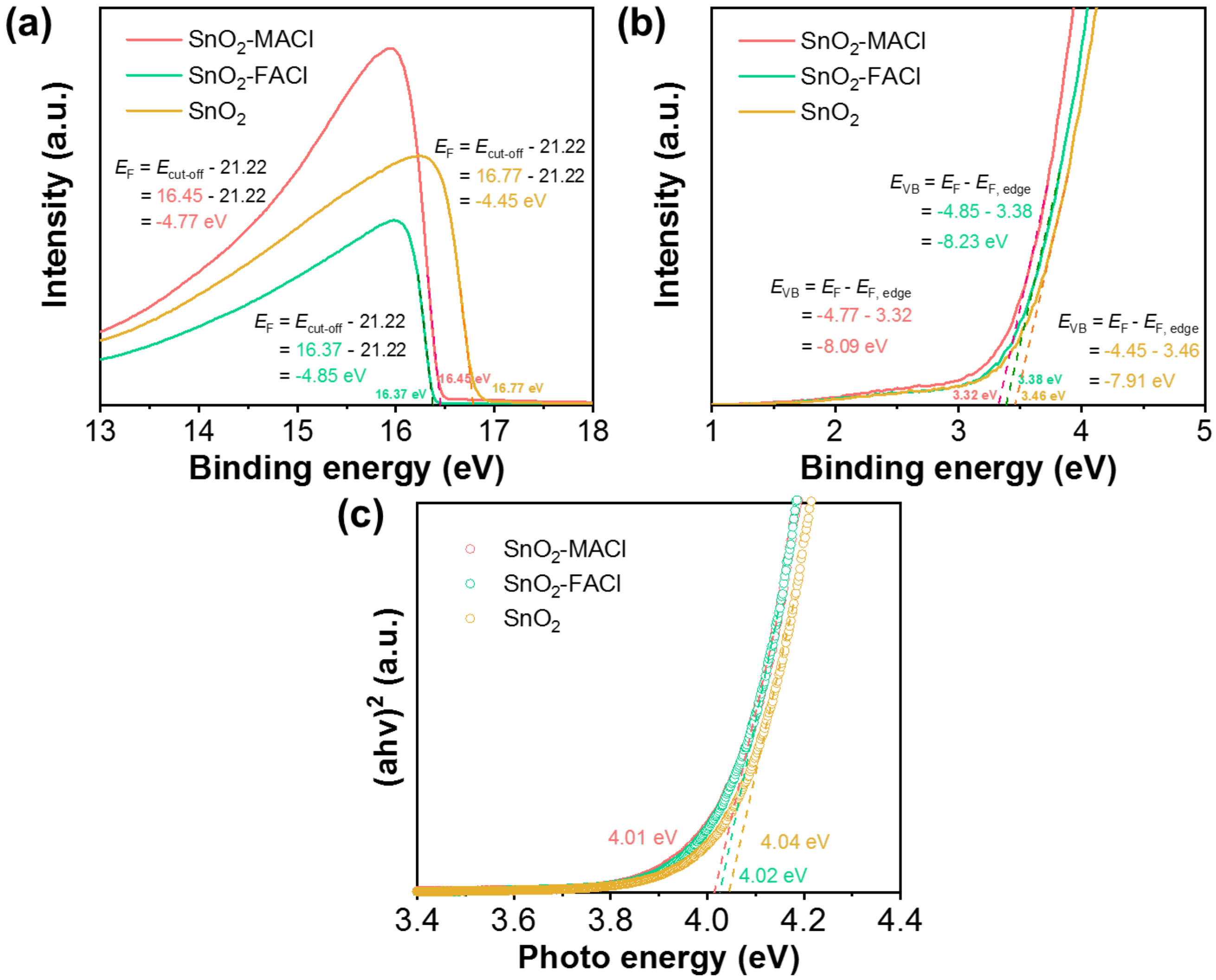

Figure S4. UPS (a and b) and UV-Vis (c) spectra of the SnO_2-MACl, SnO_2-FACl, and pure SnO_2 films.

© 2021 Wiley-VCH GmbH

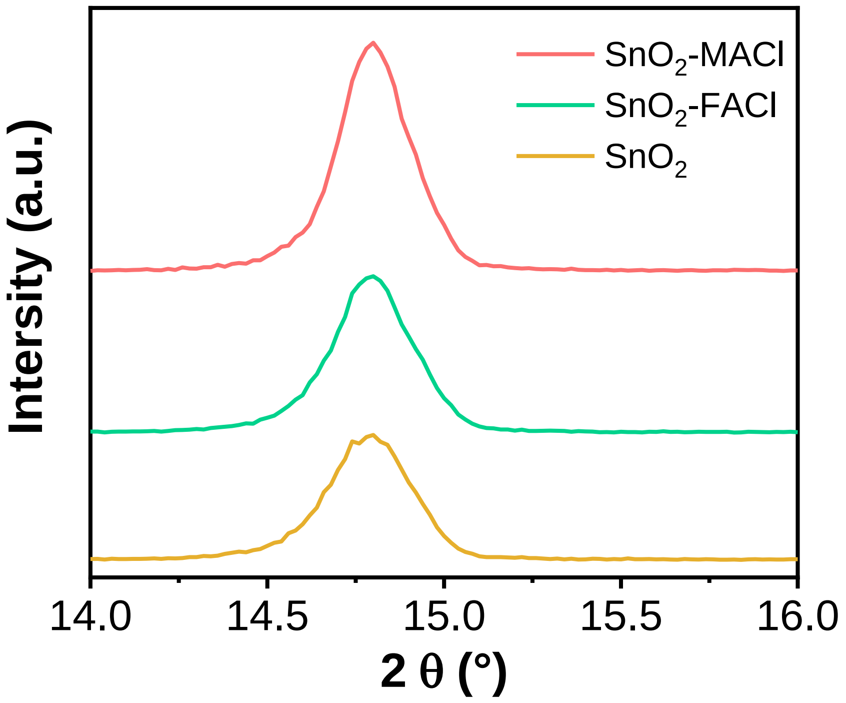

Figure S5. (110) peak in XRD patterns of perovskite films deposited on the SnO_2-MACl, SnO_2-FACl, and pure SnO_2 films.

© 2021 Wiley-VCH GmbH

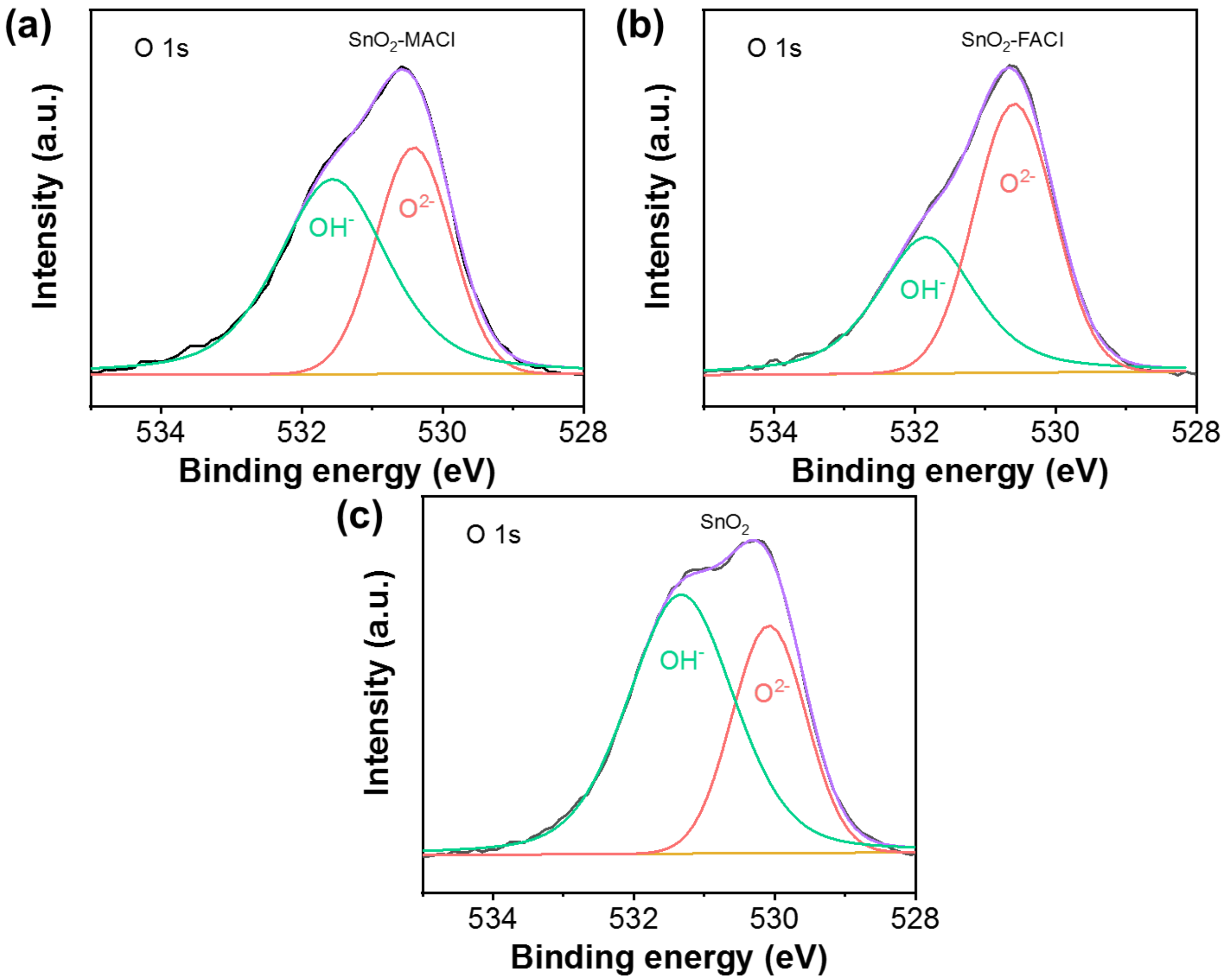

Figure S6. XPS curve of a) SnO_2-MACl, b) SnO_2-FACl, and c) pure SnO_2 ETLs at O 1s level.

© 2021 Wiley-VCH GmbH

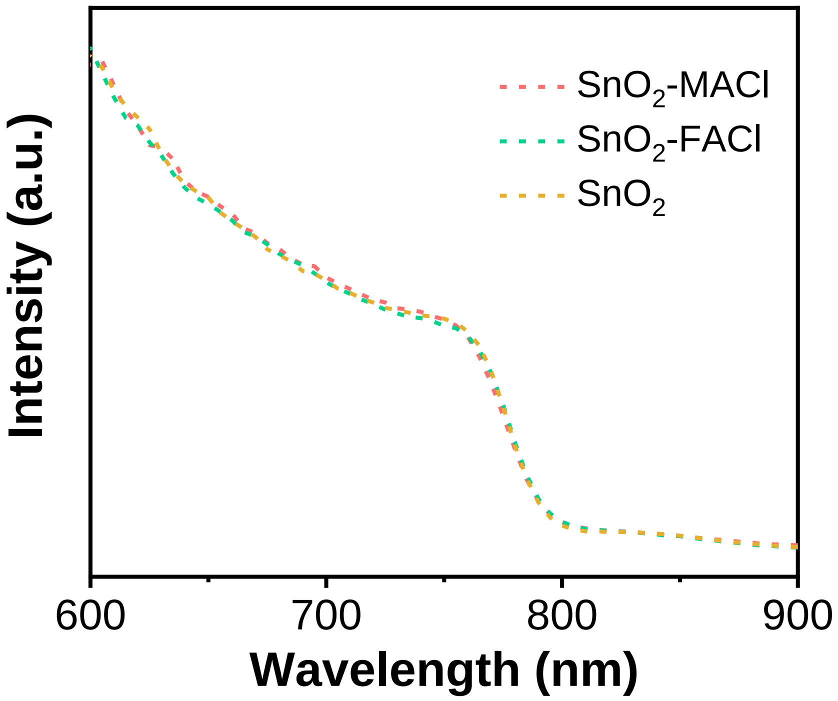

Figure S7. UV-vis absorption spectra of the perovskite film on SnO_2-MACl, SnO_2-FACl, and pure SnO_2 ETLs.

© 2021 Wiley-VCH GmbH

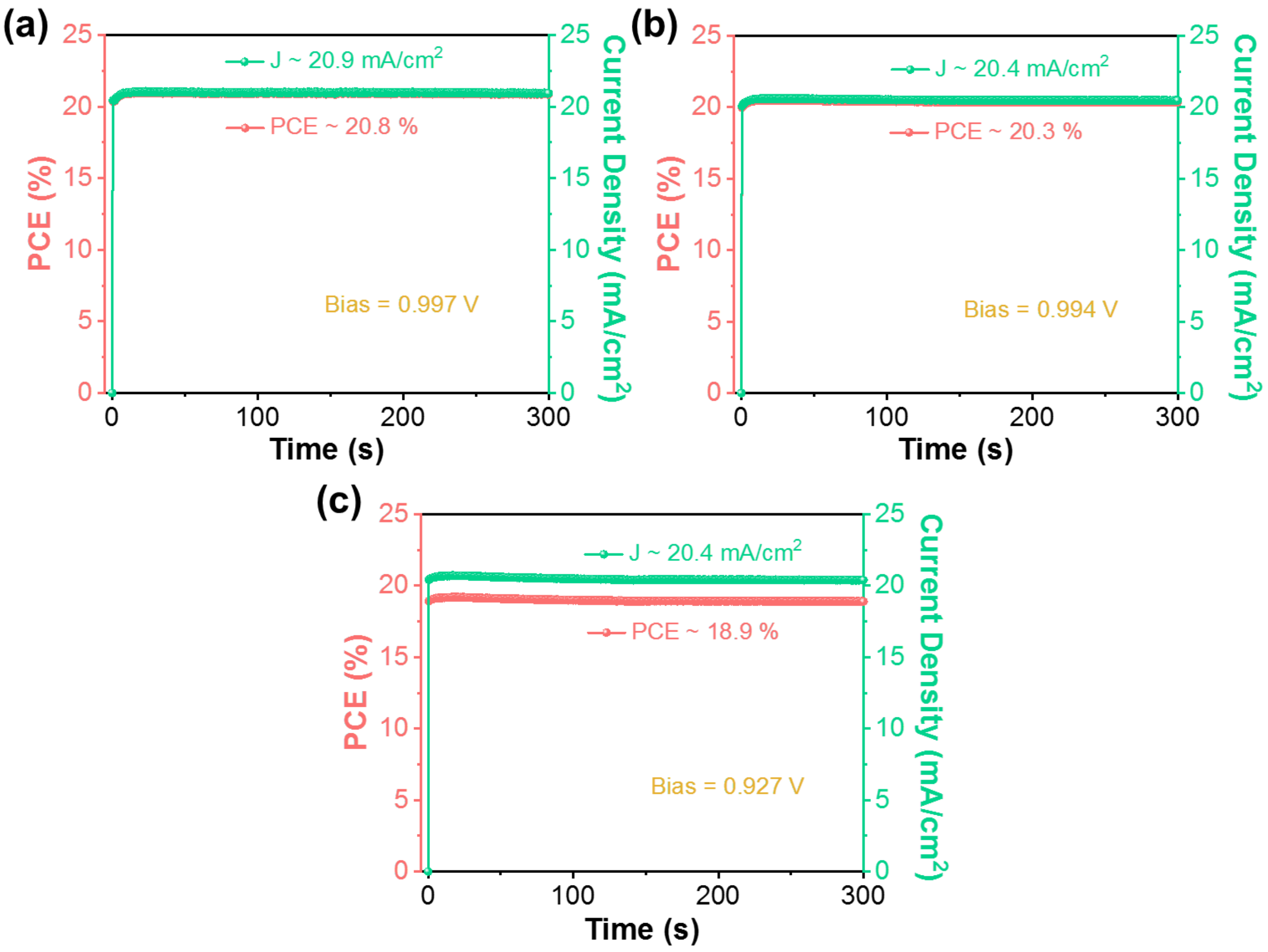

Figure S8. Steady-state efficiencies and photocurrent densities of the PSCs based on a) SnO_2-MACl, b) SnO_2-FACl, and c) pure SnO_2 ETLs.
